# Genetic Basis of Dilated Cardiomyopathy in Dogs and Its Potential as a Bidirectional Model

**DOI:** 10.3390/ani12131679

**Published:** 2022-06-29

**Authors:** Karen R. Gaar-Humphreys, Talitha C. F. Spanjersberg, Giorgia Santarelli, Guy C. M. Grinwis, Viktor Szatmári, Bernard A. J. Roelen, Aryan Vink, J. Peter van Tintelen, Folkert W. Asselbergs, Hille Fieten, Magdalena Harakalova, Frank G. van Steenbeek

**Affiliations:** 1 Department of Cardiology, Division Heart & Lungs, University Medical Center Utrecht, Utrecht University, 3508 GA Utrecht, The Netherlands; k.r.gaar-humphreys-3@umcutrecht.nl (K.R.G.-H.); t.c.f.spanjersberg@uu.nl (T.C.F.S.); f.w.asselbergs@umcutrecht.nl (F.W.A.); m.harakalova@umcutrecht.nl (M.H.); 2 Regenerative Medicine Center Utrecht, University Medical Center Utrecht, Utrecht University, 3584 CT Utrecht, The Netherlands; b.a.j.roelen@uu.nl; 3 Department Clinical Sciences, Faculty of Veterinary Medicine, Utrecht University, 3584 CL Utrecht, The Netherlands; g.santarelli@uu.nl (G.S.); v.szatmari@uu.nl (V.S.); j.p.vantintelen-3@umcutrecht.nl (J.P.v.T.); h.fieten@uu.nl (H.F.); 4 Department Biomolecular Health Sciences, Faculty of Veterinary Medicine, Utrecht University, 3584 CL Utrecht, The Netherlands; g.c.m.grinwis@uu.nl; 5 Department of Pathology, University Medical Center Utrecht, Utrecht University, 3508 GA Utrecht, The Netherlands; a.vink@umcutrecht.nl; 6 Department of Genetics, University Medical Center Utrecht, Utrecht University, 3508 AB Utrecht, The Netherlands; 7 Institute of Cardiovascular Science, Faculty Population Health Sciences, University College London, London WC1E 6BT, UK; 8 Health Data Research UK and Institute of Health Informatics, University College London, London WC1E 6BT, UK

**Keywords:** cardiovascular, fibrofatty infiltration, attenuated wavy fibers, canine induced pluripotent stem cells, human induced pluripotent stem cells

## Abstract

**Simple Summary:**

Heart disease is a leading cause of death for both humans and dogs. Inherited heart diseases, including dilated cardiomyopathy (DCM), account for a proportion of these cases. Human and canine patients with DCM suffer from an enlarged heart that can no longer pump efficiently, resulting in heart failure. This causes symptoms or clinical signs like difficulty breathing, irregular heartbeat, and eventually death. The symptoms or clinical signs of this disease vary in age of onset at the beginning of symptoms, sex predisposition, and overall disease progression. Despite the many similarities in DCM in both species, only a few candidate genes so far have been linked to this disease in dogs versus tens of genes identified in human DCM. Additionally, the use of induced pluripotent stem cells, or engineered stem cells, has been widely used in the study of human genetic heart disease but has not yet been fully adapted to study heart disease in dogs. This review describes the current knowledge on the genetics and subtypes of naturally occurring DCM in dogs, and how advances in research might benefit the dog but also the human patient. Additionally, a novel method using canine engineered stem cells to uncover unknown contributions of mistakes in DNA to the progression of DCM will be introduced along with its applications for human DCM disease modeling and treatment.

**Abstract:**

Cardiac disease is a leading cause of death for both humans and dogs. Genetic cardiomyopathies, including dilated cardiomyopathy (DCM), account for a proportion of these cases in both species. Patients may suffer from ventricular enlargement and systolic dysfunction resulting in congestive heart failure and ventricular arrhythmias with high risk for sudden cardiac death. Although canine DCM has similar disease progression and subtypes as in humans, only a few candidate genes have been found to be associated with DCM while the genetic background of human DCM has been more thoroughly studied. Additionally, experimental disease models using induced pluripotent stem cells have been widely adopted in the study of human genetic cardiomyopathy but have not yet been fully adapted for the in-depth study of canine genetic cardiomyopathies. The clinical presentation of DCM is extremely heterogeneous for both species with differences occurring based on sex predisposition, age of onset, and the rate of disease progression. Both genetic predisposition and environmental factors play a role in disease development which are identical in dogs and humans in contrast to other experimental animals. Interestingly, different dog breeds have been shown to develop distinct DCM phenotypes, and this presents a unique opportunity for modeling as there are multiple breed-specific models for DCM with less genetic variance than human DCM. A better understanding of DCM in dogs has the potential for improved selection for breeding and could lead to better overall care and treatment for human and canine DCM patients. At the same time, progress in research made for human DCM can have a positive impact on the care given to dogs affected by DCM. Therefore, this review will analyze the feasibility of canines as a naturally occurring bidirectional disease model for DCM in both species. The histopathology of the myocardium in canine DCM will be evaluated in three different breeds compared to control tissue, and the known genetics that contributes to both canine and human DCM will be summarized. Lastly, the prospect of canine iPSCs as a novel method to uncover the contributions of genetic variants to the pathogenesis of canine DCM will be introduced along with the applications for disease modeling and treatment.

## 1. Introduction

Cardiac disease is a leading cause of death for both humans and dogs, and genetic cardiomyopathies account for a significant proportion of these cases [1,2]. Although information regarding the incidence rates of specific heart diseases in dogs is limited, dilated cardiomyopathy (DCM) is the second most common heart disease in large dogs with a prevalence rate as high as up to 1 in 2 individuals in some breeds [3], and it is estimated to be the third most prevalent inherited form of heart disease in humans with a prevalence rate of approximately 1 in 250 individuals [4,5].

DCM presents similarly in humans and dogs in terms of disease phenotype and progression, which makes canine DCM a promising animal model to study human DCM [1,6]. At the same time, progress in research made in human DCM can have a positive impact on the care given to dogs suffering from DCM. Currently, the majority of animal models that exist to study the molecular consequences and cellular progression of DCM are induced models as opposed to naturally occurring DCM in dogs [7]. There are many advantages to studying naturally occurring canine DCM cases in which no external factors are needed to induce the disease phenotype. Unlike in other laboratory animal models, DCM in canines correctly models the effects of aging, which is a strong risk factor to developing heart disease [8,9]. Additionally, the complex genetic background of disease is better represented and since these animals are kept as companion animals, the environments in which they live are identical. A comprehensive One Health approach which leads to a better understanding of DCM as a whole has the potential to lead to better overall care and treatment for all patients as well as the potential for improved selection for breeding and disease prevention in canines.

On the other hand, in humans, extensive research has been done using induced pluripotent stem cells (iPSCs), a type of pluripotent stem cell that can be generated from a somatic cell. iPSCs provide an unprecedented opportunity to study the pathogenic mechanisms of inherited cardiomyopathies in vitro [10]. In recent years, the genes involved in human DCM and the mechanisms in which a disease phenotype develops have been studied by establishing human-iPSC-based cell models [11]. The same prospect exists for dogs by extending this new and rapidly developing technology to veterinary medicine. Based on similar disease phenotypes between dogs and humans, applications of discoveries can be used to the advantage of both species.

This review will corroborate the feasibility of canines as a naturally occurring, bidirectional disease model for DCM. The distinct histopathological subtypes of canine DCM will be described by comparing control and DCM tissue of multiple breeds, and the relevance to human DCM will be discussed. The known genetics that contribute to both canine and human DCM will be summarized. Lastly, the prospect of canine iPSCs (ciPSCs) as a method to uncover contributions of currently unknown genetic defects to the pathogenesis of DCM will be introduced along with the applications for human DCM disease modeling and treatment.

## 2. Clinical Aspects of Canine and Human DCM

In both dogs and humans, DCM is often characterized by ventricular dilation and systolic dysfunction, and the clinical signs and symptoms are directly related to the degree of disease severity [1,12]. Patients are at a significantly higher risk for developing congestive heart failure, ventricular arrhythmias and possible sudden cardiac death [13,14].

Overall, the clinical presentation of DCM is very heterogeneous. Differences occur based on sex, age of onset, rate of progression, and both genetic predisposition and environmental factors play a role in developing a unique phenotype [15,16]. In dogs, breed-specific differences often occur with the disease phenotype being more homogenous within each breed.

Some clinical signs of DCM, in humans and dogs, include dyspnea, cough, exercise intolerance, weight loss (cardiac cachexia), ascites, syncope, and loss of appetite. Upon examination, patients may present with tachypnea, dyspnea, and arrhythmia including atrial fibrillation, ventricular premature complexes, and ventricular tachycardia confirmed using an ambulatory electrocardiogram or 24-h Holter-monitoring. Treatments for human DCM patients focus on managing the clinical manifestations of heart failure and arrhythmias, including pharmacological treatment, device therapy, and heart transplantation [17]. The treatment for dogs is similar to humans with the goal to minimize the effects of heart failure using drugs including diuretics, angiotensin-converting enzyme inhibitors, positive inotropes, and other vasodilators [1,18,19].

A diverse array of causes for DCM have been identified for both humans and canines including genetic mutations [1,20], infection and autoimmunity [21,22,23,24], toxin exposure like alcohol and cytostatics [25,26], metabolic dysfunction and nutritional deficiencies [26,27,28], and even pregnancy [24,29]. Nevertheless, idiopathic and familial DCM are among the most frequently reported causes [12]. The availability of diagnostic tools, such as genetic testing and cardiac imaging, varies greatly around the globe. With extensive genetic testing becoming more readily available, there would possibly be a decrease in the prevalence of idiopathic DCM due to more precise diagnoses and genetic mapping [30]. Most familial DCM is inherited in an autosomal dominant pattern, but all inheritance patterns have been found, including X-linked, autosomal recessive, and mitochondrial DNA-linked [30,31].

DCM has multiple phases of progression, beginning with a long asymptomatic period. This asymptomatic period is known as the occult phase, and during this time there are no outward signs or symptoms, yet cardiovascular electrical and structural changes can be seen using an ECG and echocardiography, respectively [6,32]. In dogs, this phase can last up to a few years and for humans, this could take decades. During the final stage of DCM, patients suffer from most of the clinical signs of congestive heart failure with a poor prognosis.

## 3. DCM Histopathology

**Figure 1:**
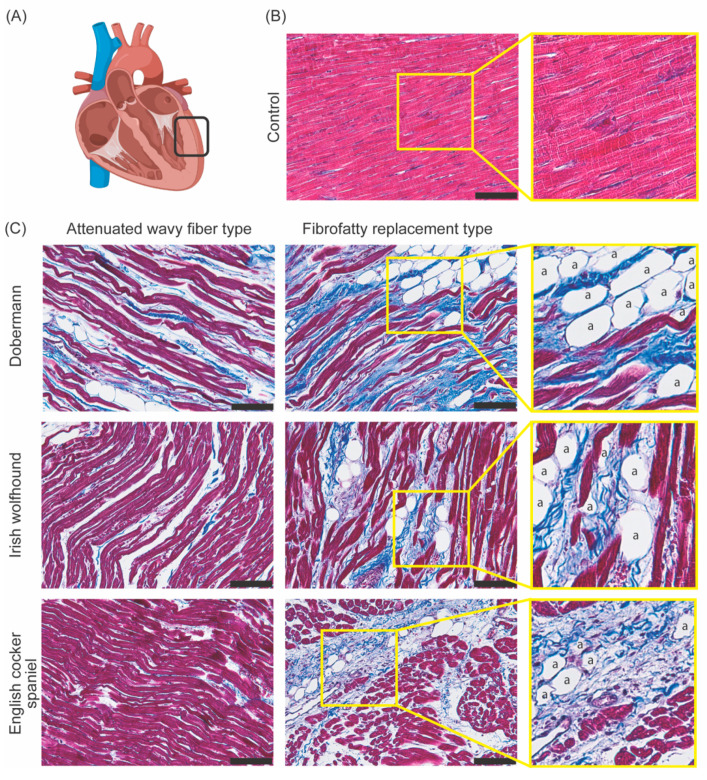
Histologically distinct forms of canine DCM. Masson’s trichrome staining of 4 µm thick paraffin embedded slides [39]. (Red = cardiomyocytes, blue = fibrotic/connective tissue, a = adipocyte, black scale bar = 100 µm). (**A**) Model of the heart showing the location (depicted in the black box) of the collected tissue in the left ventricle. (**B**) Control tissue (3-year-old male Beagle with no cardiac symptoms) showing healthy cardiomyocytes. (**C**) Diseased tissue (4-year-old male Dobermann, 5-year-old Irish wolfhound, and 7-year-old English cocker spaniel) showing the attenuated wavy fiber type and fibrofatty type in three pathologically confirmed DCM cases.

Associated with differences in survival time after diagnosis, histology of cardiac tissue, inheritance patterns, and age of onset, there is considerable evidence to suggest that there are distinct types of DCM in both dogs and humans [33,34,35]. In healthy control hearts, there is a homogenous myofiber pattern with very few inconsistencies. However, in dogs with DCM, two distinct histological myofiber patterns have been documented: attenuated wavy fiber type and fatty infiltration type (Figure 1) [13,33]. The fatty infiltration type has previously only been reported in Dobermanns, Estrela mountain dogs, Great Danes, and Boxers, and is known to affect both the right and left side of the heart. In humans, this is referred to as fibrofatty replacement and is mostly observed in patients with arrhythmogenic cardiomyopathy. Patients, both human and canine, with the fatty infiltration type of myofiber pattern may show myofiber degeneration, myocytolysis, and cardiomyocyte atrophy [33]. Typical histopathological characteristics found in this pattern include vacuolar degeneration of myofibers, atrophic myofibers, fatty infiltration, and increased collagen deposition [33,36]. In unpublished work, we observed the presence of fatty infiltration in the Irish wolfhound and additionally in the English cocker spaniel.

The attenuated wavy fiber type has been documented in many breeds, although still mostly giant-breed dogs, and primarily affects the right atrium and ventricle. It has even been found concurrently with the fatty infiltration type [1,31,37]. This type is mostly characterized by consistent myofiber atrophy and is documented in both dogs and humans [38]. The presence of the wavy attenuated fibers has been suggested as an early sign of DCM development, and the significantly decreased size of the wavy myofibers (<6 µm compared to 10–20 µm in healthy controls) results in a left ventricular wall that can be up to half the normal thickness of a healthy control [33]. We do not exclusively see one or the other fiber type, but rather the tissue is incredibly heterogeneous with both fiber types existing in different areas of the tissue in the same dog.

Distinct fibrosis patterns in human genetic cardiomyopathies have been identified [39]. Heart tissue from patients with mutations in different cardiomyopathy genes showed distinct patterns and the posterolateral left ventricle wall appeared to be the most discriminative region between different mutation carriers [39]. Varying levels of subendocardial fibrosis, interstitial fibrosis, replacement fibrosis, and fibrofatty replacement contribute to the varying histopathological patterns of each mutation. Different degrees of adipocyte infiltration in the genetically distinct human DCM subtypes has been proven [39]. Like humans, canine genetic cardiomyopathies are expected to follow these unique and specific histopathological patterns.

There is some evidence to suggest that the histological form of DCM could even be linked to survival time, which for dogs diagnosed with DCM can vary from days to several years [33]. Although ECG data, age of onset, dyspnea, and other signs of heart failure can be linked to survival, interestingly, neither breed nor left ventricular function were identified as significant factors affecting prognosis [33]. However, the low survival rate of Dobermanns (3%) could indicate that the fatty infiltration type of DCM carries a worse prognosis than the attenuated wavy fiber type, as this breed typically presents with a significant proportion of the fatty infiltration fiber type [33]. The exact effect on clinical outcome of each histological type has not been fully defined, and the phenotype can only be determined when analyzing tissue samples postmortem.

In purebred dogs, significant inbreeding is present and specific genetic mutations may have a high frequency within a breed. This is reflected in the histopathological patterns of DCM that are recognized in specific dog breeds. For example, Great Danes were reported to show the fatty infiltration type, the attenuated wavy fiber type, or even a combination of both, while Dobermanns and Bull Mastiffs were previously reported to show the fatty infiltration type [13,40]. One dog breed does not resemble the entire complexity of all canine DCM cases, and this presents a unique opportunity for modeling as there are multiple breed-specific models for DCM with less genetic variance than human DCM. Our findings suggest a more heterogeneous myofiber pattern for each breed with both the attenuated wavy fiber type and fibrofatty infiltration type in most dog cases of DCM. Differences in the percentages and location of each type per breed could be a possibility. More detailed analyses of the myofiber patterns in large cohorts of dogs with genetic DCM should be performed to elucidate the exact histopathological patterns per breed.

## 4. Breed-Specific Canine DCM Genetics

When comparing the known genetics behind human DCM to canine DCM, it is evident that there is much more to be discovered about the genetic background of DCM in dogs. Based on recent research focusing largely on single breeds affected by specific subtypes of cardiomyopathy, breed-specific genetic variants have been discovered using genome-wide association studies (GWAS) and candidate gene studies [41]. Many different loci have been identified, most of them on different chromosomes, which highlights the complexity of canine DCM development [1]. The identification of the genetic mutations that lead to canine DCM is important for the future of selective breeding to produce unaffected offspring as well as for the development of new therapeutic interventions.

### 4.1. Dobermanns

As of today, two known mutations in different genes are linked to DCM in Dobermanns. The first involves a 16-base pair deletion at the donor splice site of an intron 10 in the pyruvate dehydrogenase kinase 4 (*PDK4*) gene [42]. Pyruvate dehydrogenase 4 is an important regulatory protein for energy metabolism in cardiomyocytes. Cardiomyocytes prefer fatty acids as the primary source of energy instead of glucose, and this protein enables the oxidation of fatty acids by preventing glucose oxidation [42]. Although expression levels were not changed by this mutation, the function of the PDK4 protein was altered, leading to energy-deficient cardiomyocytes due to the reduction of efficient fatty acid oxidation. An in vitro study on PDK4-deficient fibroblasts from affected Dobermanns showed a markedly reduced metabolic flexibility during glucose starvation [43,44]. The inheritance pattern for this mutation is autosomal dominant and has between 60–68% penetrance [42].

The second known mutation linked to Dobermann DCM is a single missense variation in the titin (*TTN*) gene which causes a change in amino acid from glycine to arginine [45]. Titin contributes to the contraction of the heart muscle through the unfolding and folding of its many domains in response to tension and is what connects myosin to the Z-discs of sarcomeres. It is the largest protein in the body [41]. This mutation is associated with Z-disc active streaming along with decreased active tension [45]. Further research needs to be done to uncover the exact pathophysiology of this specific mutation regarding the changes in protein folding. Mutations in this breed have a prevalence of up to 58% and the presence of both mutations in a single affected dog has been identified [46,47] (p. 4).

### 4.2. Boxers

Boxers have a form of adult-onset cardiomyopathy that has been named “boxer cardiomyopathy.” A GWAS revealed a mutation in the striatin gene (*STRN*) as a cause for the development of this disease. The mutation was found to be both heterozygous and homozygous in a pedigree analysis, however, the Boxers homozygous for the mutation had significantly more arrhythmias and presented with clinical signs at an earlier age [40]. Not all Boxers that were diagnosed had a mutation in the *STRN* gene, implying that other mutations are likely to be present within this breed as well [40].

### 4.3. Irish Wolfhound

The prevalence of DCM in the Irish wolfhound is consistently documented between 24% and 29% [48,49,50] with males often being more affected than females and at a younger age [48,51]. A sex-dependent dominant gene model is postulated to play a role in disease progression in Irish wolfhounds, but not in a monogenic model [49]. The mode of inheritance for this breed is autosomal dominant but with a low level of penetrance inferring multiple factors could influence inheritance and progression and explaining why there is a relatively mild DCM phenotype observed [1]. A variety of single nucleotide polymorphisms (SNPs) that are linked to the development of DCM have been identified on six different chromosomes. Three are found in known genes: *ARHGAP8*, *FSTL5*, *PDE3B*. The SNP on chromosome 37 is located between the *MOGATI* gene and the *ACSL* gene and the SNPs located on chromosomes 1 and 17 are between introns [52].

### 4.4. Welsh Springer Spaniels

A R9H mutation in the phospholamban (*PLN)* gene was recently identified in a pedigree of Welsh springer spaniels who had a high incidence of left ventricular dilation, arrhythmia, and early sudden cardiac death [53]. This mutation in the *PLN* gene causes a single base pair change from G to A which results in an amino acid switch from arginine to histidine [53]. Disease progression is caused by decreased calcium uptake in the cardiomyocyte by SERCA2A/ATP2A2. All dogs with the mutation developed a phenotype, meaning the penetrance of the mutation is very high in dogs. Interestingly, this exact mutation has been found in humans but with a significantly lower penetrance [54].

### 4.5. Portuguese Water Dogs

It has been shown that Portuguese water dogs can inherit a fully penetrable autosomal recessive mutation that causes a juvenile form of canine DCM. A genome-wide linkage study was able to identify a locus linked with the disease on canine chromosome 8. The locus does not contain genes that have been associated with cardiomyopathy in humans yet, making it an interesting locus to further study for human DCM [55].

## 5. Genetics of Human DCM

Most cases of human DCM are still ruled as idiopathic, but approximately 20–35%, with some estimates closer to 50%, are linked as familial DCM and can be traced to a genetic variant [56,57]. Unfortunately, only in about 30% of families with familial DCM, the causative genetic mutation has been identified, leaving the remaining affected families with an unidentified or idiopathic familial DCM genetic variant [58]. The most common heredity pattern is autosomal dominant, but there are many other patterns regularly seen including autosomal recessive, X-linked, and mitochondrial although this last pattern is comparatively more rare [56]. Like canine DCM, the clinical phenotype, even within familial DCM, is incredibly heterogeneous. In addition to the major disease-inducing genes, the variable penetrance and expression of the disorder, as well as different sex susceptibilities, suggest that modifier genes could affect the severity and onset of DCM [59].

The affected genes that lead to a clinical DCM phenotype often encode for a variety of proteins that are heavily involved in the structure and broad cellular function of the cardiomyocyte (Table 1). In total, mutations associated with human DCM have been validated in about 50 different genes, yet so far 19 have moderate, strong, or definitive evidence for disease-gene association [60,61]. Some clear genotype–phenotype relationships have emerged that also have clinical implications, although recommendations for treatment are not based solely on the genetic variant [62]. Distinguishing pathogenic or likely pathogenic variants from nonpathogenic gene variants remains challenging due to the vast variation in the human genome. Additionally, the relationship between environmental and genetic factors requires further analysis to fully understand the genetic background of DCM progression [12].

Overall, the entire genetic field of DCM-inducing genetic cardiomyopathy is complex and still only partially understood. Functional analyses using both cell and animal models can lead to an enhanced interpretation of the overall consequences of genetic sequence variants, and iPSC-derived cardiomyocytes present as an unprecedented gene-specific pathophysiological model for cardiomyopathies.

In the Netherlands, there are prevalent human founder mutations that are associated with DCM. Founder mutations refer to mutations that arose in a population generations ago and were passed down from generation to generation. They can be further divided into classes: mutations in genetic isolates including large families, mutations not in isolates but with a regional distribution, and mutations that are shared with descendants of Dutch emigrants [64]. The recurrent and founder mutation p.Arg14del in the *PLN* gene was studied in a large population-based cohort study to determine the geographical distribution and origin of this specific mutation [65]. This mutation, which likely originated 575–825 years ago in the northern part of the Netherlands, is the most frequently identified mutation for Dutch cardiomyopathy patients [65]. Like with studying breed-specific mutations in dogs, founder mutations in humans enable a focused study between genotype and phenotype as well as phenotypic variability.

## 6. The Onset of DCM

DCM can be divided into two main subsets: juvenile-onset and adult-onset, with both subsets found in humans and canines [41].

### 6.1. Juvenile-Onset DCM

The annual incidence of juvenile-onset DCM, in human patients less than 17 years old, is less than adult-onset with an annual incidence rate of ≈1:170,000 in the United States [42] and 1:140,000 in Australia [43]. Unfortunately, although the incidence rate is significantly lower when compared to adult-onset DCM, the outcome for patients is significantly worse [44,45].

Like in humans, early-onset DCM can be found in certain breeds of dogs. Most notably in juvenile Portuguese water dogs [66]. After diagnosis, these dogs do not live longer than about seven months due to rapidly developing heart failure. A juvenile form of DCM with high morbidity was also found in six Dobermann puppies from one litter and in one puppy from an unrelated litter [67]. Congestive heart failure occurred in all affected puppies between 10 and 17 weeks old [67]. In human juvenile-onset DCM there are a variety of observed inheritance patterns including autosomal dominant, recessive, and X-linked, but when studying pedigrees of juvenile Portuguese water dogs, it is reported to only be inherited in an autosomal recessive pattern [46].

### 6.2. Adult-Onset DCM

The annual incidence rate for human adults developing DCM is approximately 5.5:100,000 and the average age at onset is between 20–50 years [46,47]. There is a large variation as to when human individuals present with clinical signs, and the same is true for dogs. For example, the Great Dane’s mean age of onset is 4.8 years [34], which is similar to the onset of DCM for Irish wolfhounds at 4.4 years [49], while the mean age of onset for Dobermanns is 7.3 years for males and 8.6 years for females [35]. This variation in age contributes to the heterogeneity of DCM clinical presentation. It is important to note that the life expectancy for canines varies per breed and is linked to body size [68]. When comparing the onset of disease for small versus larger dogs, this difference in biological age should be considered, although DCM effects mostly large or giant-bred dogs.

Both juvenile and adult-onset DCM result in remodeling of the myocardium. Pro-inflammatory cytokines and changes of adhesion molecules on the endothelium signal the recruitment of leukocytes which induces an inflammatory reaction [13]. Anti-inflammatory cytokines follow to counter the changes and restore homeostasis through myofibroblast activation and extracellular matrix (ECM) deposition. However, this reparative phase also induces redundant fibrosis formation in chronic conditions which we see directly in the pathology of the disease [13].

## 7. Sex Differences in DCM

In addition to the initial cardiomyopathy-inducing mutation, it is established now that other factors including environmental stimuli, age, and sex play an important role in the penetrance of the mutation and consequently the severity of the phenotype [69].

In humans, despite having an inheritance pattern of autosomal dominant in most familial DCM cases, males are significantly more often affected than females with a reported prevalence of about 60% [69]. Recent studies have documented that women have a better long term prognosis than males [70], clinical signs occur significantly earlier in males than in females [71], and that males have a greater prevalence of clinical signs and a higher mortality rate than females [72,73].

In dogs, similar sex-differences have been reported. In a complex segregation analysis of DCM in Irish wolfhounds, male dogs were significantly more often affected than females [49]. The prevalence of DCM in a group of 412 Dobermanns was found to be 58% with an equal sex distribution but males showed earlier echocardiographic changes than females [46]. This shows that the males had more early-onset DCM than females who suffered from more late-onset DCM. Further research is needed to fully understand the sex-related differences we see in canine and human DCM prevalence, etiology, and phenotype expression.

## 8. Disease-in-a-Dish

The discovery of the ability to reprogram human somatic cells into embryonic stem cell-like iPSCs has unequivocally advanced the scientific field of stem cells. Importantly, this new technique has paved the way to create patient-specific hiPSC lines to study disease mechanisms in incredibly personalized ways [74]. At a functional level, iPSCs mimic the patient-specific phenotype, allowing a personalized study of disease mechanisms as well as tailor-made therapeutic options [75]. Some well-documented advantages to iPSC research are the ability to expand almost indefinitely and to differentiate into all embryonic cell types, identical genetic identity to the patient whom the sample was derived from, and bypassing the technical issues associated with acquiring patient heart tissue and cell samples [74,75]. The iPSC techniques also eliminate the ethical issues surrounding the use of embryonic stem cells [74,75]. Taking this one step further, recent advances in tissue engineering have allowed for the design and construction of hiPSC-cardiomyocyte-based cardiac tissue-engineered constructs with a variety of therapeutic implications [76,77].

**Figure 2:**
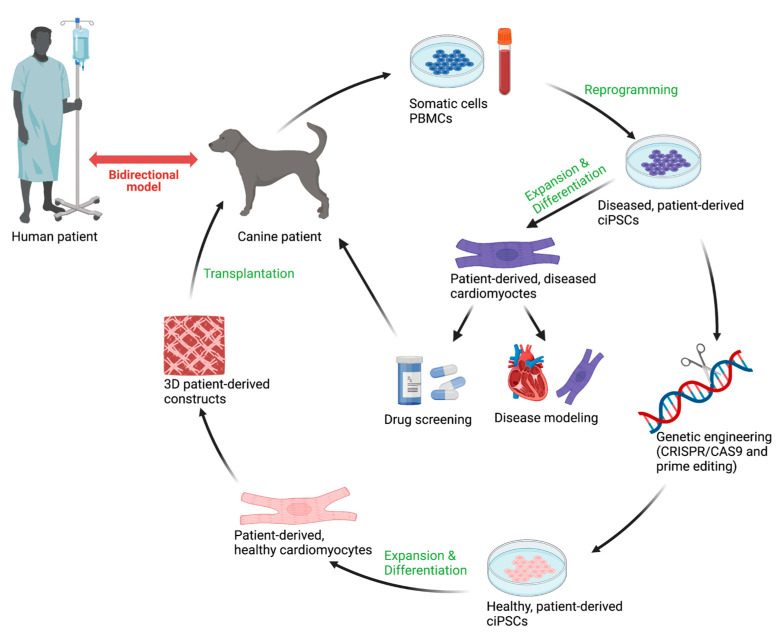
Strategy for applying canine induced pluripotent stem cells (ciPSCs) as a bidirectional model for genetic cardiomyopathies. Patient-specific ciPSC-cardiomyocytes can be used for both drug screenings and disease modeling. With the additional application of genetic engineering, healthy, patient-derived ciPSCs can be created and used for more advanced therapeutic modeling purposes. Advances made in the canine and human fields can be translated across species boundaries. Created with BioRender.com.

Induced PSC technology has been widely adopted in the field of human genetic cardiomyopathy but not yet for dogs [78]. Because of the similarities between canine and human DCM at both a histopathological and genetic level as well as similarities in disease progression, a mutation-focused canine iPSC model will be useful for genetic cardiomyopathy in both species (Figure 2).

Human iPSCs can also be used for canine research if the mutation is located within a conserved amino acid residue between dogs and humans. However, given the differences between human and dog genomes, it would be preferred to use the most similar genome. Each dog and human patient should ideally have their own derived model to account for the whole genomic variation. Recent research on human and canine epigenetic clocks demonstrates that at the DNA level there are significant similarities in the aging process, which is relevant to aging as a risk factor to DCM, further showing the translational potential of this bidirectional model [79]. Human iPSCs can now be matured for over 180 days and the chronological effects of the disease can be studied over time. Depending on the stage of the disease wanting to be studied, the amount of time the cardiomyocytes are cultured would differ.

### 8.1. Reprogramming Strategies in ciPSCs

**Figure 3:**
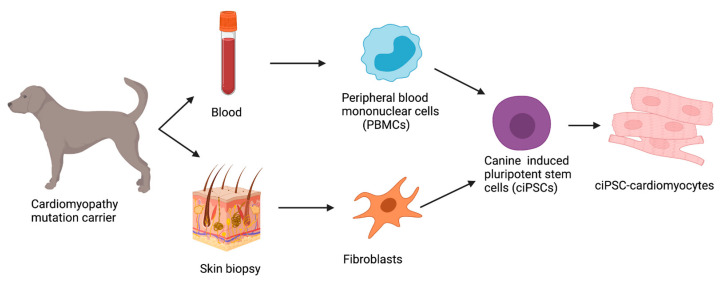
Overview of reprogramming and differentiation methods to create canine induced pluripotent stem cell cardiomyocytes (ciPSC-cardiomyocytes). The ciPSCs can be derived from both whole blood samples and skin biopsies through reprogramming. Following differentiation and expansion into cardiomyocytes, genetic cardiomyopathies can be directly modeled in a dish. Created with BioRender.com.

The first published study detailing the generation of ciPSCs was in 2010 using canine embryonic fibroblasts and lentiviral transduction with canine *OCT4*, *SOX2*, *KLF4*, *c-MYC* (OSKM) reprogramming factors [80]. Today, reprogramming strategies can successfully be applied to both peripheral blood mononuclear cells (PBMCs) collected from whole blood samples as well as fibroblasts obtained from skin biopsies (Figure 3).

Since the first successful attempt at reprogramming fibroblasts, several reports have described the generation of ciPSCs [78,80,81,82,83,84,85,86,87], but the canine cell reprogramming protocols remain inconsistent and not well established [83]. Additionally, species–species differences involved in the reprogramming, development, and maturation of iPSCs need to be further elucidated. Variations in both the reprogramming methods and the combinations of transcription factors used to create an ideal cocktail to create ciPSCs can be seen within the literature as optimization is still required (Table 2).

Initial protocols followed retroviral and lentiviral transgene integrating methods and the transgenes were eventually silenced after the iPSCs were fully reprogrammed. Due to concerns over the clinical applications with evidence from numerous studies showing that viral integrating methods can induce genomic integration and increase tumorigenic potential [99,100], other non-integrating methods have been extensively studied over the last few years. Two studies recently successfully reprogrammed canine somatic cells into ciPSCs [84,85] and one study reprogrammed canine PBMCs into ciPSCs using the Sendai viral transduction method [96]. This method is effective but much more expensive than other methods and the Sendai virus vectors can impair the regulation of reprogramming gene expression [97]. The non-viral derivation of transgene-free iPSCS from somatic fibroblasts of a male beagle dog using integration-free episomal vectors [98] and a previously reported optimized induction medium was recently successfully performed [101]. Lately, cells have been reprogrammed using an RNA transfection-based method, which is reportedly more reproducible and efficient for reprogramming [87,97]. In this strategy, canine fibroblasts were cotransfected with non-infectious, self-replicating, and integration-free Venezuelan equine encephalitis (VEE) RNA virus replicon that expresses four reprogramming open reading frames, as well as *B18R* mRNA to inhibit the immune response to VEE RNA [97]. This VEE replicon has no potential problems associated with genomic DNA integration, since it does not use a DNA intermediate, and is easier to produce than the Sendai virus particles since it requires a lower biosafety level in the laboratory [97].

Another factor still being optimized in the reprogramming protocols is the combination of transcription factors being used. Both human and mouse reprogramming factors are used for iPSC derivation in animals, and in dogs it is still unknown which reprogramming factors are the best combination. Canine OSKM factors have been used to derive ciPSCs [98], but the majority of groups reportedly use human reprogramming factors [81,88,89,91]. One reprogramming factor used, *c-Myc*, is a proto-oncogene that can induce tumor formation, although it increases the reprogramming efficiency [102,103,104]. Some recent approaches have opted to use an alternative factor, *Glis1*, which was found to promote the reprogramming of somatic cells into iPSCs more fully than the *c-Myc* gene even and decreases the risk of tumorigenicity [97,105]. This alternative factor, *Glis1*, has been reported to be used in combination with nine other factors (*OCT3/4*, *KLF4*, *SOX2*, *L-MYC*, *LIN28*, *NANOG*, *KLF2*, *KDM4D*, and *mp53DD*) [98]. It is still unclear which combination of factors and from which species is optimal for the reprogramming of canine cells.

### 8.2. Challenges in ciPSCs

Unlike in mice and human pluripotent stem cells, little is known about the final pluripotent stage of ciPSCs due to the limited number of available lines. In a recent study, ciPSCs were found to exhibit characteristics of both the naive and primed state of pluripotency and it was proposed that they belong to an intermediate state in comparison with human and mouse iPSCs [92]. Since ciPSCs are still in the early days of discovery and application, it is unknown if canine reprogramming follows the exact trajectory of human or mouse iPSCs [106]. A few barriers remain before applying ciPSCs for broader clinical use including reproducibility, the difficulty to remain in long-term culture [81,91,93,97], and the low efficiency of reprogramming. Further research is necessary to create a more efficient and reproducible reprogramming strategy for ciPSCs and to overcome the challenges seen with long-term culture.

## 9. Discussion

As of today, much more is known about the causative genes for human DCM than for dogs. This could be due to the small sample size of the available dog studies, potentially more complex inheritance patterns involving the interactions of specific genes, or combinations of variants in multiple genes and environmental factors [40]. Current research for DCM in both dogs and humans has been focused mostly on variations in the coding regions of the DNA. However, this is no longer the most accepted theory as it is widely documented that DCM in dogs and humans is inherited in a more complex pattern, so possible variants in intergenic and non-coding regions, polygenic risk scores, multiple rare and common variants, and other combinations should be additionally investigated.

The inbreeding of dogs over time for specific appearance and behavior markedly reduced the genetic diversity of certain breeds leading to an increased risk for complex disorders including genetic cardiomyopathy [107]. Today, inbreeding is still commonly used to create certain dog pedigrees leading to a high prevalence of certain mutations within one breed, and as a result, allele frequencies vary widely between breeds. Because of the smaller genetic background found in inbred dogs, there is less variance creating a unique opportunity to study genetic causative variants with less genetic diversity than in human populations. Additionally, one could argue that rather than having one canine model for human DCM, in fact there are multiple breed-specific models for different and precise phenotypes of genetic DCM creating more specific modeling capabilities.

So far, only four overlapping mutated genes are involved in the pathogenesis of DCM in both dogs and humans: *PLN*, *TTN*, *DMD*, and *RBM20*. Titin (*TTN*) mutations found in Dobermanns and mutations in phospholamban (*PLN*) in Welsh springer spaniels are already similarly identified in human patients, allowing for a model to study the pathophysiology, penetrance, and symptoms observed in these specific phenotypes. In addition, Dobermanns have already been proposed as a model for human DCM based on the similar symptoms, inheritance, and mutation between species [47]. Dystrophin (DMD) mutations are associated with X-linked Duchenne muscular disease in German short-haired pointers [1]. DMD is common in dogs and DMD-DCM is late onset in canines like humans rather than early onset as seen in mouse models. RBM20 mutations were highly associated with DCM and up to an 80% shortened life span in Standard Schnauzers [63]. Loss of RBM20 causes an abnormal intracellular calcium handling, which may relate to the arrhythmogenic presentation of RBM20 cardiomyopathy [108]. The generation of ciPSC models with these genetic variants would allow us to characterize the effects of the mutations at a deeper cellular level, compare the pathogenesis between dogs and humans, and test translatable therapies.

Although research regarding iPSCs has made many advancements this last decade, there are still some important limitations to keep in mind when considering this model including genomic instability, interline variability, genetic mutations arising during the reprogramming process, and epigenetic memory loss during cell passages [109]. The production of ciPSC lines has been inefficient and slow compared to human and mouse iPSC lines, and more attention should be paid to non-integrative reprogramming methods with a higher capability for clinical translation as well as a robust characterization of the resultant cell lines. The ciPSCs have the potential to revolutionize the study of canine genetic cardiomyopathy in vitro with the generation of relevant control and diseased cell lines. This gives the opportunity to study specific mutations at the cellular level, unravel molecular mechanisms involved in disease progression, and directly test new therapeutic interventions. 

Because of the similarities between canine and human DCM, having properly matched DCM phenotypes based on inheritance pattern, genetic studies, histopathology patterns, and survival rate would create an excellent foundation for a bidirectional model. Once this is achieved, knowledge about canine DCM phenotypes could benefit future therapeutic advances for both dogs and humans [1]. 

## 10. Conclusions

The feasibility of canines as a naturally occurring disease model for DCM was discussed, and a novel mutation-focused iPSC model was proposed to further investigate DCM-inducing genetic mutations in dogs. Because of the high prevalence of DCM in dogs, similarities in phenotype and histology patterns compared to humans, and a smaller genetic background with less variance due to inbreeding, several dog breeds are an excellent model for human DCM. Investigating novel variants linked to DCM in dogs without causative genes could provide insight into genetic variants underlying DCM in humans. Additionally, the ciPSC model would allow for the in-depth characterization of each genetic mutation, advanced disease modeling, and the development of potential therapeutic approaches with benefits for both dogs and humans.

## Figures and Tables

**Table 1:** Comparison of mutated genes involved in the development of DCM in humans and dogs. Overlap is found in genes that encode for cytoskeleton, sarcomeric, and other unclassified proteins. Bold: overlapping genes between species.

	Canine	Human [60,61]	
Sarcomeric	***TTN*** (Dobbermann [45])	***TTN*** *MYH6* *MYBPC3* *TNNT1* *MYL3* *MYL2*	*ACTC1* *MYH7* *TNNC1* *TPM1* *TNNI3* *TNNT2*
Z-disc		*MYPN* *ANKRD1* *CSRP3* *ACTN2*	*TCAP* *NEBL*
Cytoskeleton	***DMD*** (German short-haired pointer [1]) *ARHGAP8* (Irish wolfhound [52])	***DMD*** *VCL* *DES* *ILK* *LDB3* *LAM4* CRYAB	*FLNC* *SGCD* *SGCB* *SGCA* *SGCG* *PDLIM3*
Nuclear envelope		*LMNA* *TMPO* *EMD*	*SYNE1* *SYNE2*
Sarcoplasmic reticulum	***PLN*** (Welsh springer spaniel [53]) *PDE3B* (Irish wolfhound [52])	***PLN***	
RNA binding	***RBM20*** (Schnauzer [63])	***RBM20***	
Other	*PDK4* (Dobbermann [42]) *STRN* (Boxer [40]) *FSTL5* (Irish wolfhound [52])	*SCN5A* *BAG3* *DSC2* *DSP* *PSEN1*	*ABCC9* *DSG2* *EYA4* *TAZ* *PSEN2*

**Table 2:** Overview of current strategies used in the reprogramming of ciPSCs.

Reprogramming Strategy	Method
Retroviral transduction	Viral, genomic integrating [80,81,88,89,90,91,92]
Lentiviral transduction	Viral, genomic integrating [86,93,94]
Sendai viral transduction	Viral, non-integrating [84,85,95,96]
Synthetic RNA transfection	Nonviral, non-integrating [97]
Episomal plasmids	Nonviral, non-integrating [98]
